# Dominance of *Escherichia coli* sequence types ST73, ST95, ST127 and ST131 in Australian urine isolates: a genomic analysis of antimicrobial resistance and virulence linked to F plasmids

**DOI:** 10.1099/mgen.0.001068

**Published:** 2023-07-20

**Authors:** Dmitriy Li, Paarthiphan Elankumaran, Timothy Kudinha, Amanda K. Kidsley, Darren J. Trott, Veronica Maria Jarocki, Steven Philip Djordjevic

**Affiliations:** ^1^​ Australian Institute for Microbiology & Infection, University of Technology Sydney, Ultimo, NSW, Australia; ^2^​ Australian Centre for Genomic Epidemiological Microbiology, University of Technology Sydney, NSW, Australia; ^3^​ Central West Pathology Laboratory, Charles Sturt University, Orange, NSW, Australia; ^4^​ School of Animal and Veterinary Science, The University of Adelaide, Adelaide, South Australia, Australia

**Keywords:** UTIs, ExPEC, F plasmids, pUTI89, senB, antimicrobial resistance

## Abstract

Extraintestinal pathogenic *Escherichia coli* (ExPEC) are the most frequent cause of urinary tract infections (UTIs) globally. Most studies of clinical *E. coli* isolates are selected based on their antimicrobial resistance (AMR) phenotypes; however, this selection bias may not provide an accurate portrayal of which sequence types (STs) cause the most disease. Here, whole genome sequencing (WGS) was performed on 320 *E. coli* isolates from urine samples sourced from a regional hospital in Australia in 2006. Most isolates (91%) were sourced from patients with UTIs and were not selected based on any AMR phenotypes. No significant differences were observed in AMR and virulence genes profiles across age sex, and uro-clinical syndromes. While 88 STs were identified, ST73, ST95, ST127 and ST131 dominated. F virulence plasmids carrying *senB-cjrABC* (126/231; 55%) virulence genes were a feature of this collection. These *senB-cjrABC*+ plasmids were split into two categories: pUTI89-like (F29:A-:B10 and/or >95 % identity to pUTI89) (*n*=73) and non-pUTI89-like (*n*=53). Compared to all other plasmid replicons, isolates with pUTI89-like plasmids carried fewer antibiotic resistance genes (ARGs), whilst isolates with *senB-cjrABC*+/non-pUTI89 plasmids had a significantly higher load of ARGs and class 1 integrons. F plasmids were not detected in 89 genomes, predominantly ST73. Our phylogenomic analyses identified closely related isolates from the same patient associated with different pathologies and evidence of strain-sharing events involving isolates sourced from companion and wild animals.

## Data Summary

The whole genome sequences of 320 isolates described here were uploaded to the Sequence Read Archive (SRA) in the National Centre for Biotechnology and Information (NCBI), under BioProject number PRJNA842786 with BioSample accession numbers SAMN28687489 to SAMN28687824. The whole genome sequences of 24 companion animal isolates were also uploaded to the SRA under BioProject number PRJNA858941. These 24 isolates were previously analysed in Saputra *et al*. (2017). Antimicrobial resistance in clinical *Escherichia coli* isolated from companion animals in Australia. *Vet Microbiol*.;211 : 43–50. doi: 10.1016 /j.vetmic.2017.09.014.

**Impact Statement**

Urinary tract infections (UTIs) are costly, reduce patient quality of life and can lead to the development of more life-threatening conditions like urosepsis. Extraintestinal pathogenic *Escherichia coli* (ExPEC) are the most common cause of UTIs and are often treated with antibiotics, which enhances antimicrobial resistance (AMR) evolution and selection. Genomic studies of retrospective collections of *E. coli* linked to UTI are uncommon. Knowledge of the genetic characteristics of *E. coli* that cause UTIs from retrospective collections provides insight into how AMR and virulence gene carriage changes with time and sheds light on the evolutionary mechanisms that underpin these events. AMR in Australia and worldwide is rising and the landscape of lineages present and dominating in the ExPEC population is constantly changing and influenced by geography. Therefore, it is important to apply modern technology to backdated clinical ExPEC populations to study how AMR evolves. Equally important is to identify and study local transmission events, like outbreaks, to provide better policies for prevention. This research could also provide insight into the genomic bases of uropathogenicity, and possibly future ways to counteract it. Most modern genomic epidemiological studies of ExPEC focus on antibiotic-resistant lineages, such as ST131, due to their enhanced clinical significance, but this leaves sequence types which are not as resistant poorly understood, obfuscating disease burden, limiting capacity for identification of emerging pathogens and limiting our understanding of the genomic context from which pandemic ExPEC lineages emerge.

## Introduction

Urinary tract infections (UTIs) incur an enormous cost burden to society and are the leading clinical presentation that drives antibiotic prescription [1]. In Australia, UTIs cause an estimated 69 823 annual hospitalizations [2] and cost the nation’s health system AU$909 million annually [3]. Extraintestinal pathogenic *Escherichia coli* (ExPEC) are the leading cause of UTIs and are the most frequently isolated Gram-negative pathogen globally [4]. Additionally, ExPEC are responsible for bloodstream and wound infections, neonatal meningitis, and are the most frequent cause of ventilator-associated pneumonia [5]. Of more than 13 000 *E. coli* sequence types (STs), only a small subset of pandemic lineages are responsible for the vast majority of ExPEC infections [6]. Pandemic ExPEC lineages, such as ST131, have also been central to a global increase in extended-spectrum β-lactamase (ESBL)-producing *E. coli *, as well as resistance to other clinically important antibiotic classes [4, 7, 8]. A combination of sulphonamide and trimethoprim is a standard treatment for UTIs but resistance rates to these frontline antibiotics are increasing globally, leading to the elevated use of extended-spectrum β-lactams and fluoroquinolones [9–13]. Previously we performed whole genome sequencing (WGS) on 67 trimethoprim-resistant ExPEC from patients attending a regional Australian hospital in 2006–2008 [14] and reported genes conferring resistance to extended-spectrum β-lactams, heavy metals and quaternary ammonium ions co-occurring with genes encoding resistance to trimethoprim.

The gut is a major reservoir of ExPEC, with vast numbers of these organisms shed into wastewater and diverse agricultural environments where they become exposed to frequent and often constant antimicrobial selection pressures, particularly in municipal wastewater, in food animal production and in animal faecal holding ponds. Gastrointestinal carriage of major ExPEC clonal lineages is influenced by frequent host-to-host transmission facilitated by sexual contact, international travel, contaminated food and water consumption, and interactions with wildlife, livestock and companion animals [15–19]. Constant recolonization with and across different hosts and repeated exposure to aquatic and terrestrial environments undoubtedly influence how *E. coli* acquires genetic information by horizontal gene transfer (HGT). Genetic features that enable colonization of different hosts, food, livestock, companion animals, wildlife and water sanitation practices profoundly influence pathogen biology, particularly regarding the evolution of emerging lineages, and is at the forefront of predictive infectious disease management. Much of the value inherent in studying successful pandemic ExPEC lineages lies in understanding what genetic features can be attributed to their global success, notwithstanding that biological success can evolve by diverse and often convergent paths. What is clear is that the acquisition of mobile genetic elements, particularly F plasmids [18, 20–22], phage [23] and genomic islands [24, 25], contributes to lineage evolution.

The association of F virulence plasmids, such as pUTI89 and ColV, in *E. coli* lineage evolution, host range and zoonosis is an increasingly important area of enquiry [18]. However, a detailed analysis linking the carriage of these plasmids in urinary tract isolates of *E. coli* has not been conducted. In pUTI89 and related plasmids, carriage of the *cjr* operon and the putative enterotoxin gene *senB* are considered important for virulence [26]. A study that interrogated a cohort of 34 176 *E. coli* genome sequences (2570 STs) showed pUTI89 [replicon sequence type (RST) F29:A-:B10] was overwhelmingly linked to *E. coli* sourced from humans but was almost entirely absent from 13 027 *E. coli* isolates recovered from poultry, pigs and cattle [18]. F plasmids with RST F29:A-:B10 have been associated with specific sublineages of major pandemic ExPEC lineages ST131, ST73, ST69 and ST95 [14, 18, 20]. In that same study, ColV-like plasmids were represented among *E. coli* sourced from poultry [2 327/4 254 (55 %)] but also evident in 720/4 425 (16 %) human ExPEC isolates [18]. ColV virulence plasmids are found in *E. coli* that: (i) cause extraintestinal disease in humans and poultry as well as commensal *E. coli* [18, 27–29]; (ii) are required for avian pathogenic *E. coli* to cause colibacillosis [30]; (iii) have been linked with zoonotic *E. coli* infections [18]; and (iv) display resistance to chlorine [31]. However, incomplete metadata linked with isolates deposited in public databases precludes a more thorough investigation of the association of these plasmids in *E. coli* linked to UTIs. This observation has clear implications in shedding light on a deeper understanding of the One Health aspects of *E. coli* disease.

Here we have undertaken a comprehensive phylogenomic analysis of 320 *E. coli* isolates from the urine of patients experiencing different clinical afflictions but predominantly with a uro-pathological focus (cystitis, pyelonephritis) from a single rural hospital in New South Wales (NSW), Australia.

## Methods

### Sample collection

Urine samples from patients presenting at Orange Base Hospital were collected over a 6 month period (13 May to 12 November 2006). Specimens were included if they yielded a bacterial count of >10^8^ c.f.u. l^–1^ and cell count of >10^8^ l^–1^ for white blood cells and <10^3^ l^–1^ for epithelial cells. Specimens were excluded if patients had known diabetes mellitus, diarrhoea, received antibiotic therapy in the last month prior admission or were menstruating. Using these inclusion/exclusion criteria, a total of 353 samples [326 midstream urine (MSU); 27 catheter specimen urine (CSU)] were collected from 322 patients. Urine specimens were collected as previously described [14]. Briefly, each participating physician received a protocol for urine collection and the diagnostic criteria for classification of the uro-clinical syndrome. A diagnosis of cystitis or pyelonephritis required specific manifestations, as recorded by the treating medical practitioner. Cystitis-defining manifestations included dysuria, frequent urination and/or suprapubic tenderness, without fever or loin pain. Pyelonephritis-defining manifestations included urinary symptoms with a fever of ≥38 °C and flank pain, with or without nausea/vomiting. Semi-quantitative cultures were performed on horse blood, MacConkey and chromogenic agars, followed by conventional biochemical tests. Isolates were stored in 50 % (v/v) glycerol in trypticase soy broth at −70 °C until further use.

### Whole genome sequencing and genome assembly

DNA extraction and sequencing were performed as described previously [32]. Briefly, DNA was extracted using an ISOLATE II Genomic DNA (Bioline) kit following the manufacturer’s standard protocol for bacterial cells, except for the final DNA elusion step in which DNase- and RNase-free water were used. Library preparation was performed by the UTS Core Sequencing Facility at the University of Technology Sydney, using the adapted Nextera Flex library preparation kit process, Hackflex [33]. Sequencing was performed using an Illumina Novaseq S4 flow cell, 2×150 bp, at Novogene. The quality of reads was assessed using fastp (v0.20.1) [34]. Quality control was performed using assembly-stats v1.0.1 (output in Additional File 1A, available in the online version of this article). Genomes were excluded if total length was under 3.5 Mb or above 6.5 Mb. After quality control measures, the final collection consisted of 320 *E. coli* genomes derived from urine samples collected from 292 patients (multiple samples collected from 21 patients at different time points during the study timeline; however, any clonal isolates were excluded from statistical analyses; see below). Mean N50, number of contigs and read coverage was 253239, 153 and ×68, respectively.

### Phylogeny and SNP analyses

Maximum-likelihood core genome phylogenies were built using IQtree2 (v2.0.3) [35] with extended model selection and tree reconstruction by best-fit model (-m MFP), 1000 bootstrap replicates (-bb 1000), using a core genome alignment generated by Roary (v3.13.0) [36] and the fast core gene alignment with MAFFT (-e --mafft) option. All core genome phylogenies were confirmed using both marker gene-based phylogenies produced by Phylosift using the default settings [37], and SNP-based phylogenies produced by snippy v4.3.6 (github.com/tseemann/snippy) with default settings, and recombination filtering was performed using Gubbins v2.3.4 [38] (github.com/sanger-pathogens/gubbins) with default settings. Phylogroups were determined by EzClermont [39]. SNP heatmaps were built by making pairwise SNP distance matrices of core genome alignments produced by prokka using snp-dists v0.6.3 with default settings (github.com/tseemann/snp-dists) and visualized using R v4.1.2.

### Gene screening

All isolates were annotated using the prokka pipeline [40] set for *E. coli* (--genus Escherichia --species coli), forced GenBank/ENA/DDJB compliance (--compliant), standard bacterial genetic code (translation table 11) (--gcode 11). All isolates were screened for antimicrobial resistance genes (ARGs), virulence-associated genes (VAGs), the presence of ColV markers, multilocus sequence types (MLSTs; Achtman 7 Gene) and plasmid MLST (pMLST) as previously described [18], using a pipeline available at github.com/maxlcummins/pipelord2_0. This pipeline incorporates assembly-stats v1.0.1 (github.com/sanger-pathogens/assembly-stats), fastp v0.20.1, kraken2 v2.2.1 [41], mlst v2.19.0 (github.com/tseemann/mlst) and pointfinder [42]. Assemblies with a genome size of <4.5 Mb and >6.5 Mb and N50 >30 000 were excluded. Gene screening was performed using abricate v1.0.1 (github.com/tseemann/abricate) in conjunction with the following databases: VFDB [43], CARD [44], ISfinder (isfinder.biotoul.fr/) and PlasmidFinder [45]. Genes were marked as present when detected by abricate and filtered using a custom R script (github.com/maxlcummins/abricateR) to have 90 % identity and 90 % coverage. The presence and length of the class 1 integron integrase *intI1* was determined using BLAST+ v2.8.1 [46]. Contigs harbouring class 1 integrons were further analysed in SnapGene v4.1.9 (snapgene.com) and an integron map was drawn using graphics software Krita v4.4.1 (krita.org). Regarding specific IncF plasmid type groupings – as per modified Liu *et al*. [47] criteria [47] – isolates were marked as being ColV plasmid positive if they contained a gene from four or more of the following sets: (i) *cvaABC* and *cvi*, (ii) *iroBCDEN*, (iii) *iucABCD* and *iutA*, (iv) *etsABC*, (v) *ompT* and *hlyF*, and (vi) *sitABCD* [47], with ≥95 % identity and ≥95 % gene coverage. Isolates with either ≥90 % length and identity of pUTI89 (NC_007941.1) or having IncF RST F29:A-:B10 were considered to be a pUTI89-like plasmid. Plasmid mapping was performed using a custom R script available at github.com/maxlcummins/plasmidmapR. The presence of PAI-I_CFT073_ (NC_004431-P1), PAI-II_CFT073_ (NC_004431-P2), PAI-III_536_ (X16664), PAI-IV_APEC-O1_ (NC_008563) and PAI-V_536_ (AJ617685) was scored as either present (≥90 % sequence identity and ≥95 % sequence coverage), partial (≥90 % identity and <95 % but >50 % coverage) and absent (<50 % coverage).

### Statistical analyses

To visualize the clustering of the isolates based on their virulence or resistance gene profile, gene presence/absence matrices were used in conjunction with classical (metric) multidimensional scaling (MDS) performed in R Studio using cmdscale and visualized using ggplot2 v3.3.0 (ggplot2.tidyverse.org/). To determine any statistically significant differences in gene presence–absence between UTI- and non-UTI-associated isolates, Scoary 1.6.16 [48] was used with –no_pairwise flag. Isolates from same patients with the same ST and identical ARG and VAG profiles (*n*=16) were excluded from statistical analysis in sections Antimicrobial resistance and Virulence-associated genes below.

Statistical tests were performed in R v4.1.2, chi-square to determine the difference between plasmid types and *intI1* carriage performed using a standard chisq.test, while the Pairwise Wilcoxon test with Benjamini–Hochberg *P*-value correction for multiple testing was used to compare the number of ARGs between different plasmid type groups utilizing the pairwise_wilcox_test from the rstatix (cran.r-project.org/package=rstatix) package. All tests were checked for statistical power using the pwr v1.3–0 R package (cran.r-project.org/package=pwr), and significance was reported if the power of the test was ≥0.8.

## Results

### Demography

This collection consisted of 320 *E. coli* draft genomes (mean contig size 4 6581 bp, mean genome size 5 114 541 bp) originating from isolates sourced from the urine of patients attending Orange Base Hospital (OBH), Australia, in 2006. Most isolates were sourced from patients with urinary tract disease, including kidney infections (20.6 %, *n*=66) and lower UTIs (64.7 %, *n*=207), while only five isolates (1.6 %) were from patients with sepsis. Twenty-nine (9.1 %) isolates were sourced from patients with diseases not related to the urinary tract. Isolates sourced from females dominated the collection (*n*=273) compared to males (*n*=47), with 11 patients pregnant at the time of sampling. Patient age varied from less than 1 month of age to 97 years old, with an average of 54.2 years. Fifteen (4.7 %) isolates were acquired from patients ≤3 years old and 137 (42.8 %) from patients ≥65 years old. Twenty-one patients were sampled more than once with an average isolation date difference of 38 days (range 0–156 days). Nineteen of these subsequent isolates were the same ST as the previous. Metadata for all OBH isolates is provided in Additional File 1B.

### Phylogeny

**Fig. 1.**
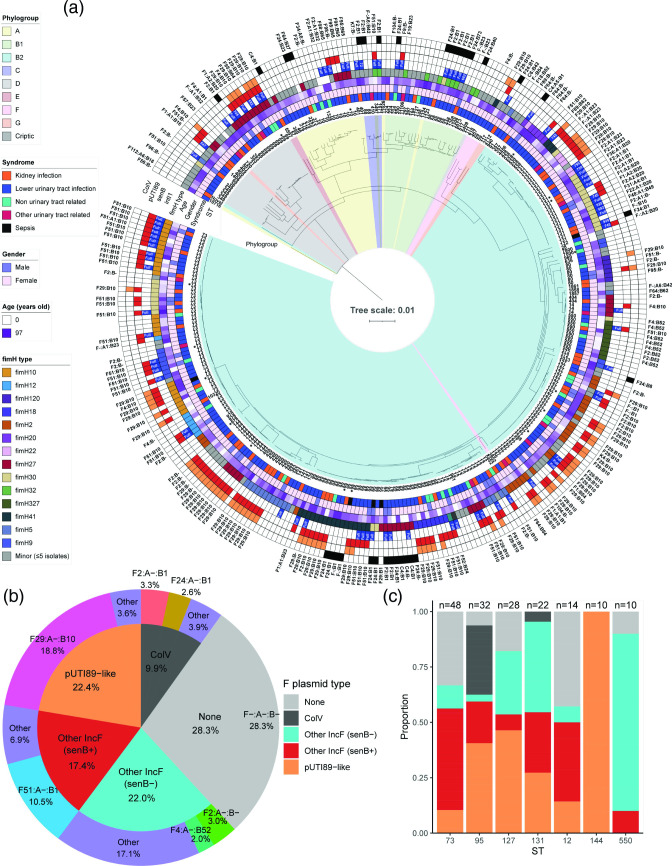
Orange Base Hospital isolate phylogeny and plasmid carriage. (**a**) Maximum-likelihood phylogenetic tree of 320 ExPEC isolates sourced from a rural hospital. Bars, the number of substitutions per site of alignment. Core genome SNP tree rooted on outgroup *Escherichia fergusonii* ATCC 35 469^T^ (not shown), including 2601 genes with a total alignment length of 2 462 224 bp, constructed by IQ-TREE2. Branch highlights coloured according to phylogroup identified by EzClermont, and leaf names represent the sequence type of each isolate. The outer ring represents the presence of the ColV plasmid according to Liu criteria [47] (black). Second outer ring: presence of pUTI89-like plasmid (orange). Third outer ring: presence of *senB* (red). Fourth outer ring: presence of *intI1* (blue) and white text over the box represents truncation length of *intI1*. F RSTs are displayed at the periphery. Isolates marked by an asterisk represent serial isolates with identical ARG and VAG profiles. (**b**) Pie chart showing the distribution of F plasmid types across the whole collection, with dark grey for ColV-positive isolates according to modified Liu *et al*. [47] criteria [47], orange for isolates positive for pUTI89-like plasmids, red for isolates positive for *senB* but negative for pUTI89-like plasmids, and blue for isolates positive for F plasmids but negative for *senB*, pUTI89 or ColV, as well as main incF RSTs present in these categories, with other for RSTs present in fewer than five isolates in particular categories. (**c**) Bar chart of proportions of the same plasmid type categories for STs with ten or more isolates.

A maximum-likelihood core genome-based phylogenetic tree (Fig. 1a) saw the isolates cluster according to Clermont phylogroup [49]. EzClermont typing showed the presence of ten clades with isolates within phylogroup B2 dominating the collection: A (*n*=23, 7.2 %), B1 (*n*=22, 6.9 %), B2 (*n*=226, 70.6 %), C (*n*=4, 1.3 %), D (*n*=26, 8.1 %), E (*n*=2, 0.6 %), F (*n*=5, 1.6 %), G (*n*=3, 0.9 %), U (*n*=8, 2.5 %) and cryptic (*n*=1, 0.3 %). Eighty-eight STs were identified. The pandemic STs dominated the collection led by ST73 (*n*=51, 15.9 %) followed by ST95 (*n*=34, 10.6 %), ST127 (*n*=31, 9.7 %), ST131 (*n*=24, 7.5 %), ST12 (*n*=14, 4.4 %) and ST144 (*n*=11, 3.4 %). Isolates with ST10, ST69 and ST550 (*n*=10, 3.1 % each), ST420 (*n*=7, 2.2 % each), ST80 (*n*=6, 1.9 %), ST349, ST538 and ST58 (*n*=5, 1.6 % each) were also notable. The most common STs associated with specific uro-clinical syndromes are presented in Table 1. ST73 with serotype O6:H1 (*n*=19) was the most prominent ST for patients diagnosed with lower UTIs. ST127 (O6:H31 *n*=5) was the most common ST in non-urinary tract-related diseases, and ST131 (serotype O25b:H4) and ST73 were frequently recovered from kidney infections. Three of the five isolates recovered from patients diagnosed with sepsis were ST95 and two carried a ColV F virulence plasmid.

**Table 1.** Most common sequence types (STs) and serotypes in isolates from different sources

Uro-clinical syndrome	Most common STs*	Most common serotypes
Kidney infections	ST73 (*n*=8), ST131 (*n*=7), ST95 (*n*=6), ST127 (*n*=5), ST144 and ST420 (*n*=4 each)	O25b:H4 (*n*=7), O134-Gp6:H31 (*n*=5), O6:H31 (*n*=5), O6:H1 and O1:H7 (*n*=4 each)
Lower urinary tract infections	ST73 (*n*=33), ST95 (*n*=21), ST127 (*n*=16), ST131 (*n*=12) and ST12/ST550 (*n*=7 each)	O6:H1 (*n*=18), O6:H31 (*n*=16), O1:H7 (*n*=10), O25b:H4 (*n*=9) and O75:H5 (*n*=8)
Non-urinary tract-related (e.g. myocardial infarction, pneumonia, migraine)	ST127, ST73 and ST12 (*n*=4 each), ST10 and ST95 (*n*=3 each)	O6:H31 (*n*=4)
Other urinary tract-related (e.g. renal failure, kidney stones, renal transplant)	ST127 (*n*=3), ST131 and 73 (*n*=2 each)	O6:H31 (*n*=3) and O25b:H4 (*n*=2)
Sepsis	ST95 (*n*=2), ST131 and ST73 (*n*=1 each)	O25b:H4, O1:H7, O2-O50-Gp7:H1 and O2-O50-Gp7:H4 (*n*=1 each)

*No statistically significant associations between most common STs and uro-clinical syndromes found.

F virulence plasmids that carry *cjrABC* together with *senB* and those that carry ColV virulence markers are associated with distinct pandemic ExPEC lineages [18, 21, 50, 51]. F virulence plasmids influence *E. coli* host range, zoonotic potential [18, 21, 32, 52] and AMR carriage in ExPEC [18, 50]. In this collection, 231 isolates carried F plasmids (72.2 %), of which 126 isolates (54.5 %) carried F plasmids with *senB-cjrABC*. These *senB-cjrABC+* F plasmids consisted of 73 pUTI89-like plasmids (62 were F29:A-:B10 and 11 were closely related to pUTI89 according to the selection criteria) and the remaining 53 were structurally different to pUTI89 [17 RSTs, predominantly F51:A-:B10 (*n*=32)] (Fig. 1b). These data highlight the dominance of *E. coli* carrying F plasmids with *senB-cjrABC* virulence genes in urinary tract disease. Notably, *senB-cjrABC*-positive plasmids were present in all 11 ST144 isolates and most ST69 (*n*=10) and ST127 (*n*=17) isolates (Fig. 1c). ColV F virulence plasmids with diverse RSTs were detected in 32 (10 %) genomes and were represented by 16 STs with ST95 the predominant lineage (*n*=12). F24:A-:B1 and F2:A-:B1 were the most common ColV RST (*n*=10 each).

*E. coli* with ST73, ST95, ST127 and ST131 accounted for nearly half (43.7 %) of the collection. Additional core genome phylogenies were constructed for each of these four STs by including publicly available *E. coli* genomes with the same ST that originated from Australia. To provide a One Health perspective, genomes from non-human host origins were included and their relatedness to our clinical isolates was determined using SNP analyses.

#### ST73 phylogeny

**Fig. 2.**
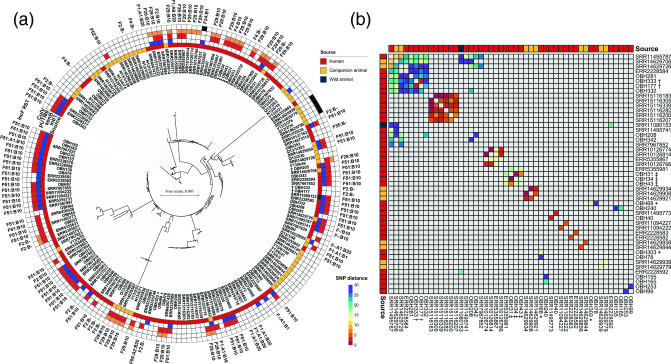
Phylogeny and SNP analysis of ST73 isolates. (**a**) Mid-point rooted core genome-based maximum-likelihood phylogenetic tree of ST73 isolates (alignment length 3 201 980 bp). Leaf names correspond to isolate names; isolate names highlighted by red colour are from this study. Inner ring represents the source of the isolates, and second ring represents the presence of *intI1* (blue). Third ring indicates the presence of *senB* (red) with the fourth ring indicating the presence of the pUTI89-like plasmid (orange). The outer ring represents the presence of the ColV plasmid according to modified Liu criteria [47] (black). Outside of the rings, F RSTs are shown at the periphery. (**b**) Pairwise SNP distance heatmap showing isolates with 30 SNPs (purple) or <30 SNPs (purple to red colour scale). Values higher than the threshold or self-comparisons are greyed out. Serial isolates are marked by *, † or ‡.

ST73 is a serologically and phylogenetically diverse ST [53]. ST73 isolates were the most abundant ST from the OBH collection. A separate core genome maximum-likelihood phylogenetic tree was reconstructed using ST73 isolates from this collection, all ST73 genomes available on EnteroBase from Australia (human *n*=84, wild animal *n*=1 and companion animal *n*=30, date range: 2006–2019; metadata in Additional File 1C), and 24 ST73 genomes from companion animals as described previously [54, 55] (Fig. 2a). This phylogenetic analysis showed that companion animal-sourced isolates tended to cluster together and that these isolates were less likely to carry F plasmid replicons (i.e. 27 % of isolates originating from humans were F plasmid negative, as opposed to 67 % in isolates from animals). Isolates from OBH were broadly distributed with no evident clustering by source or serotype (Fig. 2a). Nevertheless, we did identify multiple instances of small SNP differences in isolates originating from different hospitals as well as between clinical isolates and those derived from companion animals (Fig. 2b). Specifically, we highlighted clusters of ST73 isolates that displayed very close SNP distances: (i) an isolate retrieved from a wild animal (flying fox, SRR11080153) which differed by 27 SNPs to companion animal-sourced isolate SRR14629706 – the flying fox-sourced isolate also differed by 29 and 20 SNPs respectively to human-sourced isolates SRR11495787 and ERR2228592; (ii) two closely related companion animal-sourced isolates SRR14629706 and SRR14629726 to seven human-sourced isolates (average 22 SNPs, range 10–33); and (iii) closely related human-sourced isolates, including four OBH isolates from different patients comprising two with lower UTI, one with renal calculi and one with acute myocadiac infarct, with an average of 24 SNPs across them (range 3–29 SNPs).

We were able to identify F plasmid replicon STs in 116 (61 %) ST73 isolates depicted in Fig. 2. ST73 was dominated by F plasmids carrying *senB-cjrABC* (*n*=85, 73.3 %) with only five isolates (4.3 %) predicted to carry a ColV plasmid including a single ST73 carrying F24:A-:B1 and a cluster of four phylogenetically related isolates with an F untypable replicon ST. pUTI89 (F29:A-:B10 *n*=27) ST73 isolates were dispersed across the phylogeny. F plasmids related to pUTI89 comprised F2:A-:B- (*n*=2), F2:A-:B10 and F4:A-:B10 (both *n*=1). *senB-cjrABC* plasmids distinct from pUTI89 with F51:A-:B10 (50/85; 58.8 %) was the dominant RST. A significant number (*n*=74; 38.9 %) of ST73 genomes did not carry an F plasmid (F-:A-:B-). Liu *et al*. [47] developed criteria that have been useful in identifying *E. coli* carrying ColV plasmids [21, 47]. We noted that 98 ST73 isolates tested false positive using these ColV marker criteria many of which (*n*=55, 56 %) were F-:A-:B- by pMLST. Read mapping analyses (data not shown) failed to provide evidence of the presence of ColV plasmids in these isolates. Rather these false-positive ColV ST73 isolates carried a suite of genomic islands (see ‘Virulence-associated genes’ section below) noted for carriage of ColV virulence markers. To address this, we increased the stringency of the Liu *et al*. [47] criteria [47] to ≥95 % identity and ≥95 % gene coverage as outlined in the Methods and this removed the false positive status of these ST73 isolates.

#### ST95 phylogeny

**Fig. 3.**
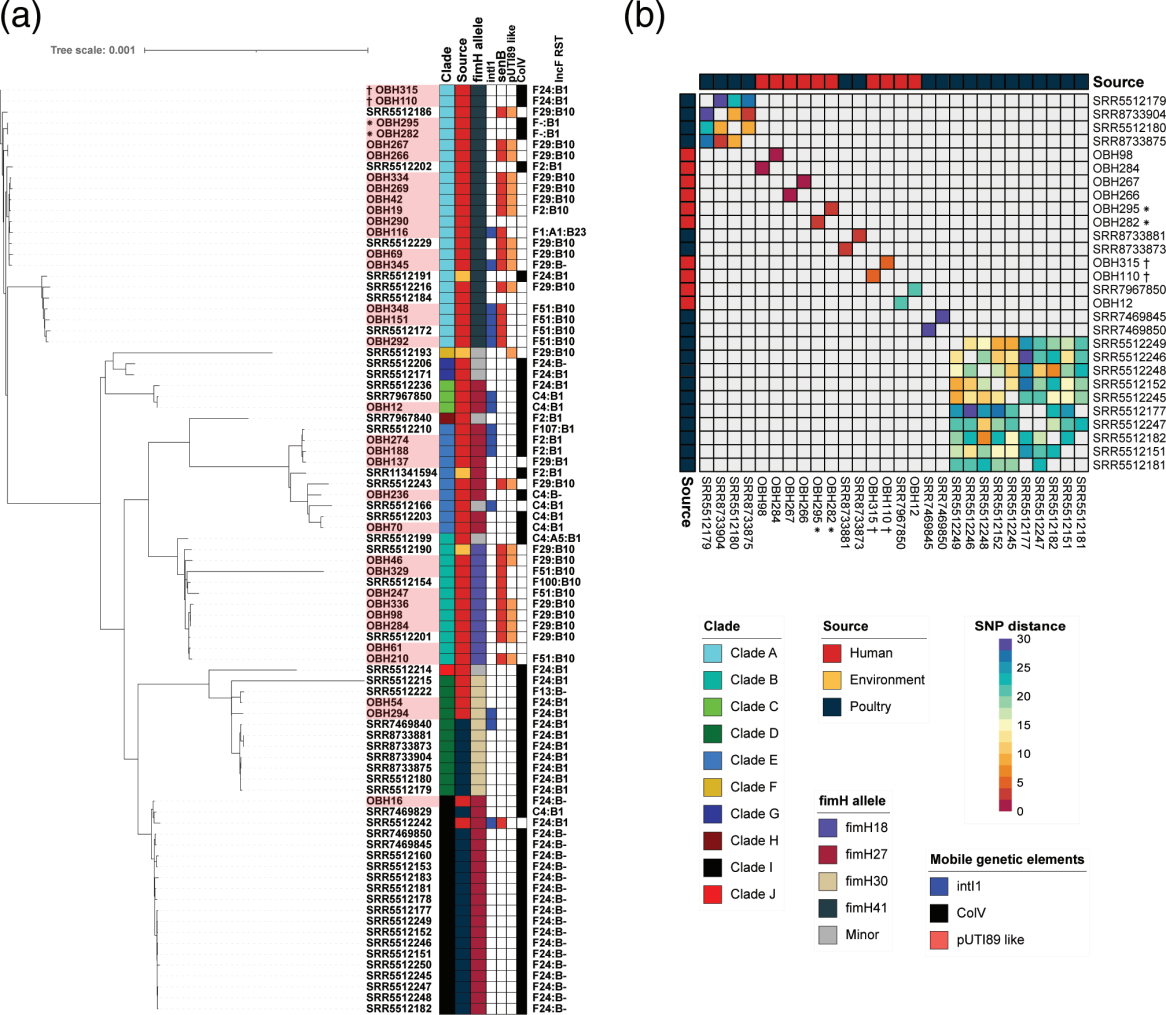
Phylogeny and SNP analysis of ST95 isolates. (**a**) Mid-point rooted core genome-based maximum-likelihood phylogenetic tree of ST95 isolates from this collection, plus 51 additional Australian ST95 isolates of varying HC20. Total alignment length: 3 448 827 bp. Leaf names correspond to isolate names; isolates from this collection are highlighted in red. Coloured squares on the right represent metadata for each isolate, first column represents ST95 clades, second refers to source, third column represents *fimH* allele, fourth presence of *intI1* (blue), fifth presence of *senB* (red), and sixth presence of pUTI89-like plasmid (orange). Outer ring represents presence of the ColV plasmid according to Liu criteria [47] (black). Outside label corresponds to F RST of the isolate. (**b**) Pairwise SNP distance heatmap showing pairs with 30 SNPs (purple) or lower (purple to red gradient); values higher than the threshold or self-comparisons are greyed out. Serial isolates are marked by * or ✝.

A core genome maximum-likelihood phylogenetic tree of 34 ST95 isolates from the OBH as well as an additional 51 genomes from EnteroBase derived from Australian ST95 sourced from poultry (*n*=25), humans (*n*=22) and the environment (*n*=4) (date range: 2001–2019; metadata in Additional File 1C) with varying Hierarchical Clustering (HC20) designations was constructed (Fig. 3a). Previously we showed that *E. coli* ST95 isolates can be categorized into ten distinct clades (A–J) [18]. A maximum-likelihood core genome phylogenetic tree (Fig. 3a) revealed that the majority of ST95 isolates from the OBH collection belong to the human-centric clade A (*n*=17, 50 %), followed by clade B (*n*=8, 24 %) and clade E (*n*=5, 15 %). No OBH isolates were identified as belonging to clades F, H or J. Small SNP differences between isolates from different sources were only observed between one human isolate and a poultry isolate (both in Clade I; 64 SNPs) (Fig. 3b). However, examples of close human-sourced ST95 isolates from clade A were found including isolates from the same patients: OBH266 (cystitis) and OBH267 (cystitis) which were collected on the same day differed by 2 SNPs; OBH282 (nephritis) and OBH295 (sepsis) were 3 SNPs apart; and OBH110 (cystitis) with OBH315 (cystitis) which were collected on the same day differed by 6 SNPs. Also present were closely related isolates from different patients: OBH284 (chronic cystitis) and OBH98 (urethritis) were 2 SNPs apart; and OBH12 (urethritis) and SRR7967850 (sepsis) were 21 SNPs apart. Notably, isolate SRR7967850 was sourced from Concord Repatriation Hospital in Sydney in 2013, approximately 7 years and over 200 km apart.

pUTI89-like plasmids were present in 13 isolates (38 %), most of which belonged to clade A (*n*=8, 61 %) with *fimH*41 and serotype O1:H7. Similarly, ColV plasmids were present in 12 isolates (35 %), but as expected most ColV-positive isolates did not belong to human-dominated clades A or B (*n*=8, 67 %) but were dominant in clades I and in clades C and E. The distribution of ColV and pUTI89-like plasmids across the major STs identified in this study can be viewed in Fig. 1(c). Notably, ST95 had the highest carriage of ColV plasmids, while other STs including ST127, ST69 and ST144 were predominantly associated with pUTI89-like plasmid carriage. Consistent with this we recently described the carriage of pUTI89-like plasmids in ST127 from canine and human origins [32].

#### ST127 phylogeny

**Fig. 4.**
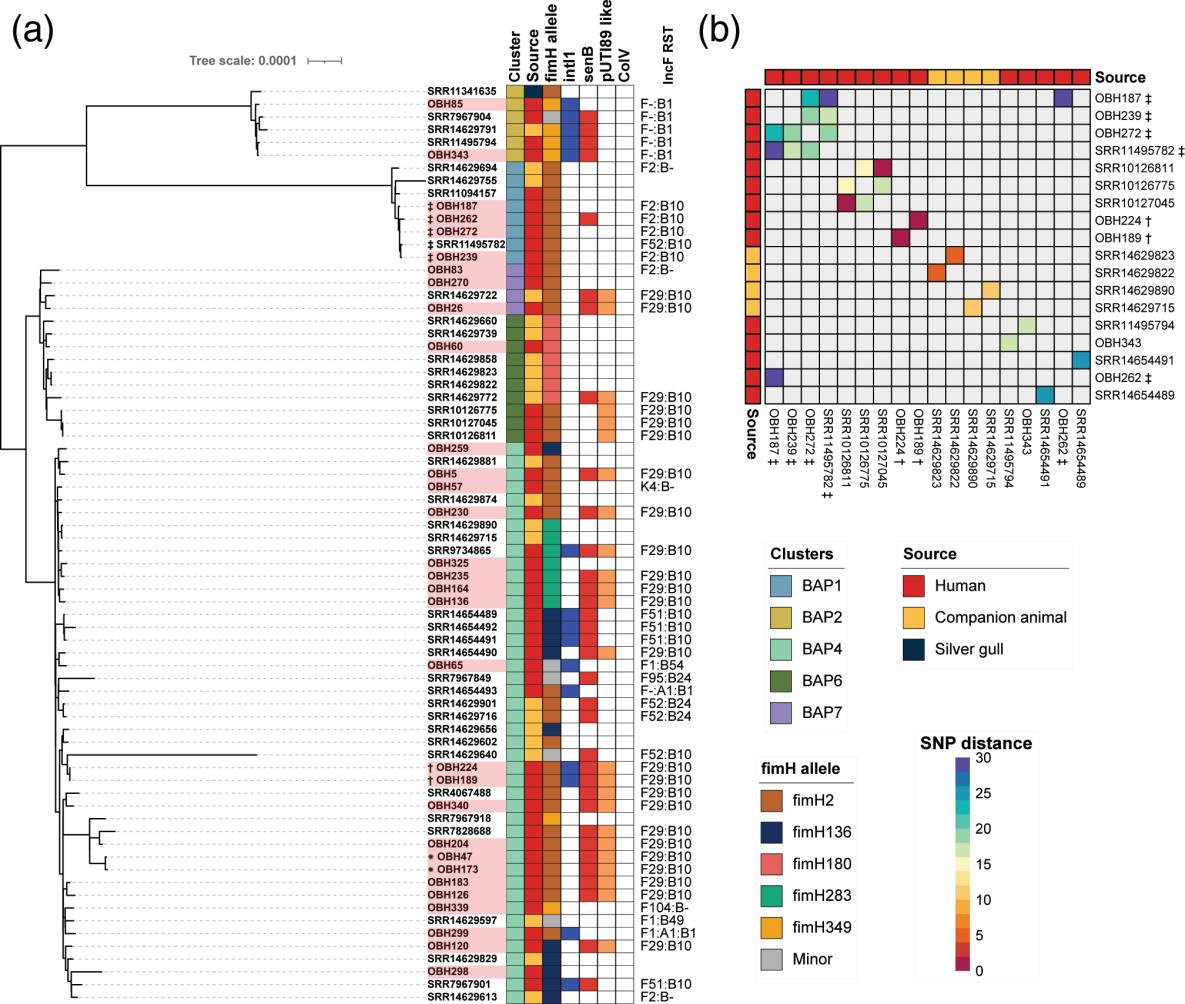
Phylogeny and SNP analysis of ST127 isolates. (**a**) Mid-point rooted core genome-based maximum-likelihood phylogenetic tree of ST127 isolates. Rooted by an ST372 isolate (SRA accession number: SRX10969655; not shown on the tree). Total alignment length of 3 579 832 bp. Leaf names correspond to isolate names, with isolates from this study highlighted in red. Coloured squares on the right represent metadata for isolate: first column represents source, second column *fimH*, third presence of *intI1* (blue), fourth presence of pUTI89 like plasmid (orange) and fifth presence of *senB* (red). Outer ring represents presence of the ColV according to Liu criteria [47] (black). Outside label corresponds to incF RST of the isolate. (**b**) Pairwise SNP distance heatmap showing pairs with 30 SNPs (purple) or <30 (purple to red colour gradient); values higher than the threshold or self-comparisons are greyed out. Serial isolates are marked by *, ✝ or ‡.

A core genome maximum-likelihood phylogenetic tree of ST127 was built using an additional 41 Australian ST127 isolates from EnteroBase (human *n*=18, companion animal *n*=22, wild animal *n*=1; date range: 2006–2019, metadata in Additional File 1C) (Fig. 4a). The 31 ST127 isolates from this collection were broadly distributed among four main clusters. Overall, ST127 isolates were found to be diverse, with 6178 SNPs separating the two most distant isolates (companion animal-sourced isolates SRR14629755 and SRR14629640). However, we did identify several closely related isolates (Fig. 4b) including: (i) four isolates, all originating from one patient of OBH (OBH187, 239, 272, SRR11495782) experiencing different uro-clinical syndromes (pyelonephritis, cystitis and glomerulonephritis) over a 2 month period, had an average of 24 SNPs (range 18–33 SNPs); (ii) two clinical isolates from different patients (OBH343, cystitis; and SRR11495794, urosepsis), were found to have distance of 18 SNPs; and (iii) two isolates, OBH189 and OBH224, isolated 24 days apart from the same patient with a recurrent UTI were indistinguishable (0 SNPs).

#### ST131 phylogeny

**Fig. 5.**
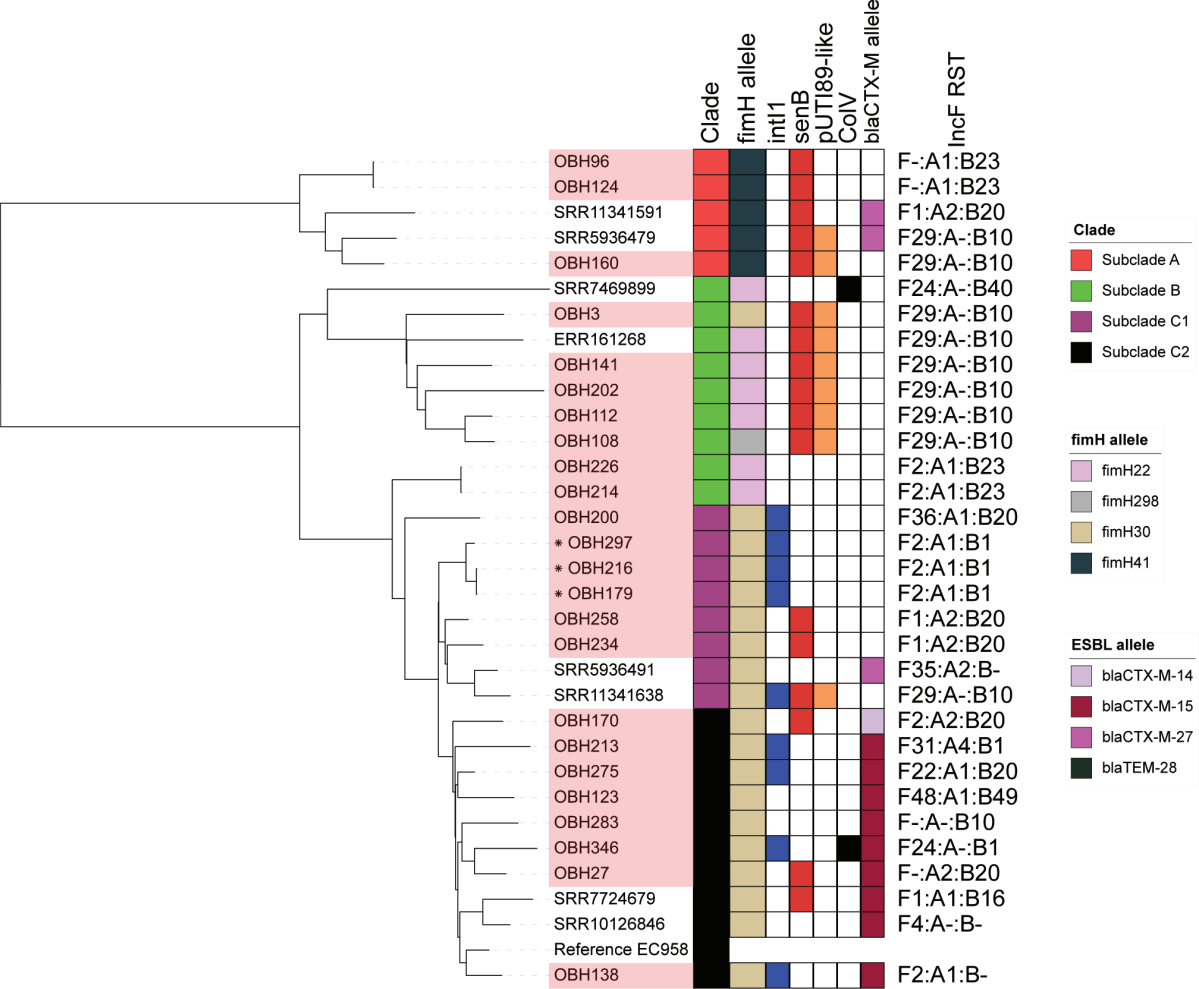
Phylogeny and SNP analysis of ST131 isolates. Mid-point rooted core genome-based maximum-likelihood phylogenetic tree of ST131 isolates. Total alignment length of 3 702 378 bp. Leaf names correspond to isolate names; isolates from the current study are highlighted in red. Coloured squares on the right represent metadata for isolates; first column: clade, second column: *fimH* alleles, third: *intI1* (blue), fourth: presence of pUTI89 like plasmid (orange), fifth: presence of *senB* (red). Outer column represents the presence of the ColV plasmid according to Liu criteria [47] (black). Outside label corresponds to incF RST. *Serial isolates.

We recently performed comprehensive genomic analyses on all available *E. coli* ST131 genomes originating from Australia (*n*=284) [56], so here we provide an SNP-based recombination filtered maximum-likelihood phylogenetic tree of ST131 isolates of this collection plus eight Australian ST131 isolates, from 2008 to 2019 (metadata in Additional File 1C), representing the major clades C1, C2, B and A [56, 57] (Fig. 5). Most isolates from this collection belonged to clade C (*n*=14, 58 %), followed by seven (29 %) in clade B and three (13 %) in clade A. The diversity of F RSTs differed among ST131 clades: OBH isolates in clades A and B carried three different F RSTs, F29:B10 (*n*=6), F2:A1:B23 (*n*=2) and F-:A1:B23 (*n*=2), and all of these were pUTI89-like plasmids. Clade C1 isolates also had three types present (F2:A1:B1 *n*=3, F1:A2:B20 *n*=2, F36:A1:B20 *n*=1) while in clade C2 every isolate (*n*=8) had a unique F plasmid RST.

ST131 isolates are known to be highly clonal, especially within the C1 and C2 clades [56], but using a 30 SNP cut-off we only found three isolates within this threshold: OBH179 (pyelonephritis), OBH216 (glomerulonephritis) and OBH297 (cystitis). These were all sourced from the same patient. OBH216 was isolated 27 days after OH179 and differed by 28 SNPs and OBH297 was isolated 15 days after OBH216 and differed by 12 SNPs. Only one ST131 isolate from our collection was found to be ColV positive (OBH346, clade C2).

### Antimicrobial resistance

**Fig. 6.**
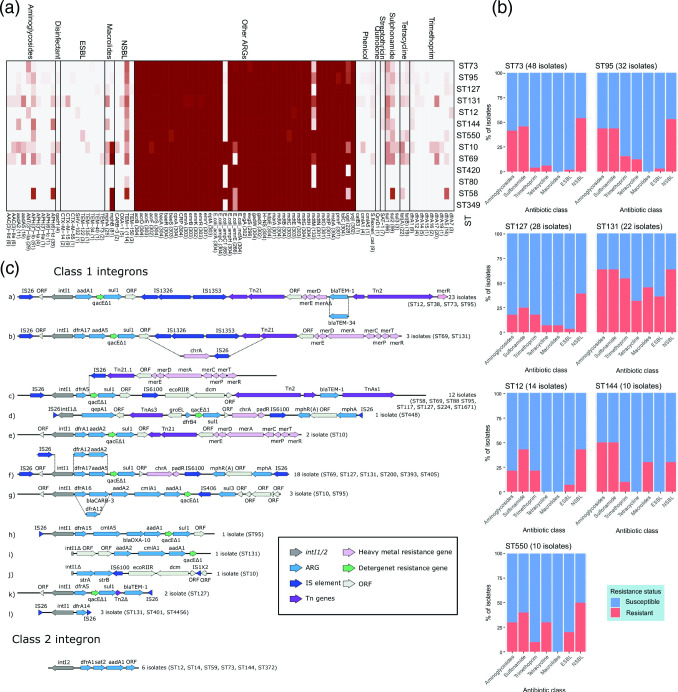
Genomic antibiotic resistance analysis. (**a**) Gene screening heat map for antibiotic resistance genes for STs with ≥5 isolates. (**b**) Bar chart of genomic antibiotic resistance in STs with ≥5 isolates. ESBL – extended-spectrum β-lactamase, NSBL – narrow-spectrum β-lactamase. (**c**) Class 1 (a–l) and class 2 integron structures identified in this collection. Integron integrase genes in grey, antimicrobial resistance genes (ARG) in cyan, insertable (IS) elements in blue, transposon (Tn) genes in purple, heavy metal resistance genes in pink, detergent resistance genes in green, and other ORF in white.

We identified 95 ARGs in this collection (Fig. 6a; Additional File 1D). Only 12 isolates (4.3 %) carried genes encoding ESBLs, and these were predominantly found in ST131 isolates (*n*=9). Only isolates in ST131 clade C carried ESBLs with seven positive for *bla*_CTX-M-15_, and one for *bla*_CTX-M-14_ and *bla*_TEM-28_ each. Sulfonamide resistance genes were found in more than a third of all isolates (37.2 %; *n*=113), and the most common gene was *sul1* (*n*=66), followed by *sul2* (*n*=59) and *sul3* (*n*=3). Similarly, genes conferring resistance to aminoglycosides were identified in 32.2 % of isolates (*n*=98), the three most common being *aph(3'')-Ib* (*strA*) (*n*=51), *aph(6)-Id* (*strB*) (*n*=35; additional 16 truncated) and *ant(3'')-IIa* (*n*=29). Genes conferring resistance to trimethoprim were found in approximately one-fifth of this collection (19 %; *n*=58), the most prevalent being *dfrA17* (*n*=20), *dfrA5* (*n*=14) and *dfrA1* (*n*=9). The recently characterized *dfrB4* gene [58] was identified in just one isolate (ST448 OBH273). Tetracycline resistance genes were identified in 41(13.5 %) isolates, with *tet(A*) (*n*=22) being most common, followed by *tet(B*) (*n*=19) and *tet(D*) (*n*=1). Only one isolate carried both *tet(A*) and *tet(B*). Genes conferring resistance to macrolides, chloramphenicol, streptothricin, quinolones and fosfomycin were less common. The macrolide resistance gene *mphA* was found in 8.2 % (*n*=25) of isolates. Genes conferring chloramphenicol resistance were found in seven isolates (2 %) (*cmlA1*: *n*=5, *cmlA5: n*=1, *catB3*: *n*=1). The streptothricin resistance gene *sat2* was found in seven isolates (two ST73, one of ST12, ST14, ST59, ST144 and ST372 each). The quinolone resistance gene *qnrD1* was identified in only one ST127 isolate. Similarly, just one ST1727 isolate carried the fosfomycin resistance gene *fosA7*.

The collection was also screened for specific point mutations in *gyrA* and *parC* genes that confer resistance to fluoroquinolones. The *gyrA*-1AB mutation was identified in 4.9 % of the isolates (*n*=15), most commonly in ST131 (*n*=11), but also in ST393 (*n*=2), ST448 and ST405 (*n*=1 each). The *gyrA*-1AB and *parC*-1aAB dual mutations were identified in 3.9 % of isolates (*n*=12): 11 in ST131 (Clades C1 and C2) isolates and in one ST405 isolate.

Overall, the carriage of ARGs was distributed as follows: 3.9 % of isolates for gentamycin resistance (based on *aac(3)-IId*, *aac(3)-IIe, aph(3’)-IIa* [59]), 37.5 % for sulfonamide resistance alone, 18.1 % for trimethoprim and sulfonamide resistance combined, 4.3 % for ESBLs, and 3.9 % for fluoroquinolone resistance. Sixty-one per cent (*n*=185) of isolates did not carry genes or mutations conferring resistance to any of these antibiotic classes and no isolates carried carbapenemase genes.

Despite the skewed frequency of ARGs overall, there were no associations between uro-clinical syndrome and ARG profiles nor any statistically significant differences between uro-clinical syndrome and the mean number of ARGs. Similarly, there were no associations between ARG profiles or the mean number of ARGs in terms of patients’ biological sex and age (Additional Files 1E and 1F). The distribution of ARGs conferring resistance to clinically important antibiotics differed among the major STs in this collection, with ST131, ST69 and ST10 isolates exhibiting robust multidrug-resistant (MDR) profiles (Fig. 6b).

Class 1 integrons can capture and express ARGs and lead to an MDR phenotype. The capture and shuffling of ARGs are mediated via the integrase *intI1* [60]. Here, a total of 80 isolates (26.3 %) contained *intI1*. Most (*n*=67) carried the non-truncated form of *intI1*. In the remaining 13 isolates, seven different *intI1* truncations were observed (Fig. 1a), the most prevalent being *intI1*_Δ745_ (*n*=4). Overall, the most common class 1 integron structure was *intI1-aadA1-qacEΔ1-sul1* associated with a Tn*21* transposon, and a Tn*2* transposon (containing the *bla*_TEM-1_ gene) inserted into the *mer* operon (mercury resistance) [Fig. 6c(a)]. This particular class 1 integron confers resistance to aminoglycosides, sulphonamides, narrow-spectrum beta-lactams and quaternary ammonium compounds. This structure was dominant in ST73 (*n*=16/23) and isolates from patients with lower UTIs (*n*=17/23). In one isolate (ST73), an SNP in the *bla*_TEM-1_ gene gave rise to *bla*_TEM-34_, thereby providing putative ESBL resistance. Only one isolate (an ST131 sourced from a kidney infection) was identified carrying two class 1 integrons [Fig. 6c(i), c(l)]. The highest prevalence of class 1 integrons was found in ST131 (54.5 %, *n*=12) followed by ST10 (44.4 %, *n*=4) and ST12 (42.8 %, *n*=6), which correlates with these STs having MDR profiles as mentioned above. There was a significant difference found between plasmid type and class 1 integron carriage (χ^2^=83.56, df=4, *P*<2.2e-16), with *senB-cjrABC*+/non-pUTI89-like plasmids (encompassing 17 IncF RSTs; most common F51:A-:B10) being most associated with *intI1*. In particular, 77 % (*n*=27) of isolates carrying F51:A-B10 were *intI1* positive (Additional File 1D). Conversely, isolates that carried pUTI89-like plasmids had significantly lower *intI1* carriage (*n*=5, 7.9 % vs *n*=78, 46.6 %, χ^2^=15, df=1, *P*=0.0001). Isolates that carried *senB-cjrABC*/non-pUTI89-like plasmids also carried on average the most ARGs associated with HGT (*n*=4.5), which was significantly different to other plasmid types (pUTI89-like *P*=1.48e-07; *senB*- IncF plasmids *P*=0.000162; no IncF plasmid *P*=1.83e-16) (Additional File 1F).

The class 2 integron *intI2-dfrA1-sat2-aadA1* was identified in six isolates (Fig. 6c), providing resistance to trimethoprim, streptothricin and aminoglycosides. Other integron classes were not detected.

### Virulence-associated genes

**Fig. 7.**
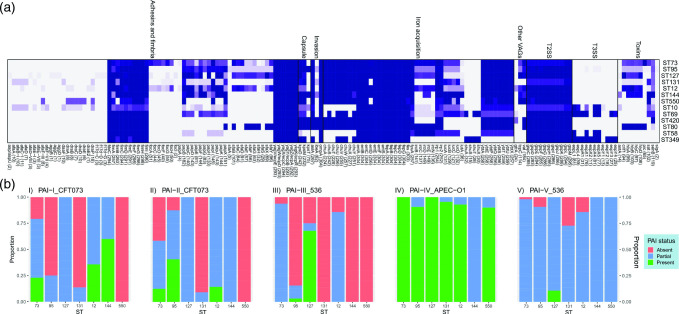
Genomic virulence analysis. (**a**) Gene screening heat map for virulence-associated genes for STs with ≥5 isolates. T2SS – type 2 secretion system, T3SS – type 3 secretion system. (**b**) Presence status of five pathogenicity-associated islands (PAIs) for STs with ≥10 isolates.

A total of 218 VAGs were identified in the collection (Fig. 7a; Additional File 1D). The molecular definition of uropathogenic *E. coli* (UPEC; a subset of ExPEC) is defined as the presence of ≥3 of the following genes: *chuA*, *fyuA*, *yfcV* and *vat* [61]. By this definition, 219 (72 %) isolates qualified as UPEC, but 75 isolates obtained from patients with UTIs did not possess ≥3 of these genes, including 60 isolates from lower UTIs and 15 from kidney infections. Notably, a scoary analysis found no statistically significant difference in VAG carriage (or any other gene) between UTI- and non-UTI-associated isolates (Additional File 1G). Similarly, no associations between the overall VAG profile and uro-clinical syndrome, sex and age were observed (Additional Files 1E and 1F). Across all age groups, sexes and pathologies, genes involved in iron acquisition, adhesion, immune evasion and toxins were widespread. Iron acquisition systems play an important role in surviving the iron-scarce environment of the urinary tract [62]. The ferric yersiniabactin uptake receptor *fyuA* was found in 85.3 % (*n*=273) of isolates. The iron-regulatory proteins *irp1* (*n*=270, 84.3 %) and *irp2* (*n*=268, 83.7 %) were similarly common, with the complete yersiniabactin siderophore operon *ybtAEPQSTYX* present in 85 % (*n*=272) of isolates. The Salmochelin siderophore system encoded by *iroBCDEN* was identified in 45.6 % (*n*=146) of isolates. The full aerobactin operons *iucABCD* and *iutA* were present in 11.6 % (*n*=37) of isolates.

Specificity for uroepithelium has been demonstrated for type 1 fimbriae and P-fimbriae which are important for UTI-causing ExPEC [62]. Type I fimbriae genes were present in most isolates and *fimH*, which encodes a protein that binds to uroepithelial associated α-d-mannosylated proteins, was present in 98.1 % of isolates (*n*=314). P-fimbriae, encoded by the *pap* operon, bind to receptors located in the upper urinary tract and are associated with nephritis [63]. However, we did not find a higher association between the presence of pap operon genes and kidney infections.

ExPEC have well-documented immune evasion mechanisms and produce toxins facilitating damage of the host tissues [64, 65]. Immune evasion mechanisms include increased serum survival protein (*iss*) present here in 74.7 % (*n*=239) of isolates, VirB5-like protein TraT (*traT*) observed in 65.6 % of isolates (*n*=210) and outer-membrane protein T (*ompT*) detected in 10 % of isolates (*n*=32). Toxins identified included enterotoxin TieB protein (*senB*) observed in 38.8 % (*n*=124) of isolates, full alpha-haemolysin operon (*hlyABCD*) present in 31.9 % (*n*=102) of isolates, cytotoxic necrotizing factor 1 (*cnf1*) found in 30.9 % of isolates (*n*=99) and heat-stable enterotoxin 1 (*astA*) identified in 4.4 % (*n*=14) of isolates.

Iron acquisition systems, adhesins and toxins are commonly found on pathogenicity-associated islands (PAIs). We screened for the presence of five PAIs (Additional File 1h) – PAI-I_CFT073_ carrying the haemolysin operon (*hlyABCD*), PAI-II_CFT073_ with P fimbriae operon (*papACDEFGHIJK*), PAI-III_536_ containing adhesion (*sfaABCDEFGHSX*) and the iron acquisition genes (*iroBCDEN*, putative haemin receptor), PAI-IV_APEC-O1_ which carries the yersiniabactin operon (*fyuA*, *irp1*, *irp2*, *ybtAEPQSTUX*), and PAI-V_536_ containing adhesins (*pixABCDFGHJ*, *pgtABCP*) and capsule-related operons (*kpsCEFMSTU*).

The presence of ExPEC-associated PAIs was scored as either present (≥90 % sequence identity and ≥95 % sequence coverage), partial (≥90 % identity and <95 % but >50 % coverage) or absent (<50 % coverage). Evidence for a complete PAI-I_CFT073_ was found in 24 isolates, mostly in ST73 (*n*=11) and ST144 (*n*=6) isolates. A partial copy of PAI- I_CFT073_ was found in 99 isolates. Most isolates (60.6 %; *n*=60) carrying a partial copy of PAI- I_CFT073_ contained the full *hly* operon but not a full *pap* operon. PAI-II_CFT073_ was identified in 22 isolates, mostly with ST95 (*n*=13), followed by ST73 (*n*=6). Nearly half (45.8 %; *n*=22) of ST73, 47 % (*n*=15) of ST95, 85.7 % (*n*=12) of ST12, and all ST127 and ST144 isolates carried a partial island, with most missing *ireA* (63.3 %, *n*=69) and *papA* (70.6 %, *n*=77). PAI-III_536_ was present in 21 isolates, almost exclusively within ST127 isolates (*n*=19), while ST73 contained the most (93.7 %; *n*=45) partial versions of this PAI followed by ST12 (85.7 %; *n*=12). Isolates carrying a partial PAI-III_536_ were missing the putative lysin/cadaverine transporter and the putative cadaverine decarboxylase genes. PAI-IV_APEC-O1_ was present in most isolates (*n*=186; 61.2 %). The first 28321 bp (36.5 %) of this PAI has 97.92 % sequence identity to the high pathogenicity island (HPI). Most phylogroup B2 isolates carried a full PAI-IV_APEC-O1_ (*n*=170) or a partial version (*n*=39). Of the 39 partial versions, all encompassed a complete HPI. Only three isolates contained a complete PAI-V_536_, all of which were ST127, but only a small proportion (14.2%, *n*=30) of phylogroup B2 isolates did not possess a partial PAI-V_536_ (all PAI maps and mosaic plots showing cross-sectional distributions of PAIs across sex, age and pathologies can be viewed in Additional File 1h).

In summary, the top six pandemic STs (ST73, ST131, ST95, ST69, ST127 and ST12) often carried the five PAIs to different degrees (Fig. 7b). PAI-IV_APEC-O1_ was present in almost all B2 phylogroup isolates. Out of all the STs, ST127 isolates had the highest proportion of carrying either complete or partial versions of all five PAIs.

## Discussion

ExPEC comprise diverse STs; however, globally only a subset is responsible for most infections. A recent systematic review found that 85 % of ExPEC infections were attributed to just 20 STs, the top five being ST131 > ST69 > ST10 > ST405 > ST38 [6]. In this study of urine-sourced clinical *E. coli* isolates (*n*=320) from an Australian regional hospital in 2006, the most common STs were ST73 > ST95 > ST127 > ST131 > ST12 which accounted for 48 % of all isolates. Most of the 20 STs flagged by Manges *et al*. (2019) [6] were represented in this collection, with the exception of ST648, ST354, ST167, ST617, ST23 and ST1193. *E. coli* ST1193 is a rapidly emerging global MDR lineage that is particularly prevalent in Australia [51]. Its notable absence in our 2006 collection suggests that ST1193 had not taken hold in ExPEC that cause UTI in Australia at this time. While ExPEC STs vary in abundance and diversity within human populations [66], our observed difference between the top ST distributions probably stemmed from the fact that most studies included in the review were biased because the collections were based on antibiotic resistance [6]. Indeed, our previous study on trimethoprim-resistant *E. coli* UTI isolates from the same hospital [14] found that the most common STs were ST131 and ST69. However, when sample selection was not based on AMR phenotypes, our current results reflect other studies from the UK, USA and Canada, wherein ST73, ST95, ST127 and ST131 were the most frequently isolated STs from large cohorts of UTIs [67–69].

Given the importance of AMR, ongoing surveillance of resistant populations undoubtedly has merit, but it does not provide an accurate portrayal of the STs most responsible for UTIs. *E. coli* ST73 as a primary causative agent of UTIs is likely to be underrepresented in the literature as the lineage is often described as pan-sensitive to antibiotics [70, 71] and plasmid naïve [72]. This was not so in this collection, as many ST73 isolates carried ARGs conferring resistance to β-lactams, sulphonamides, aminoglycosides, class 1 integron integrases *intI1*, and at least one plasmid replicon. Notably, however, only one ST73 isolate carried an ESBL resistance gene. The most common plasmid type detected in ST73 isolates was *senB-cjrABC+/*non-pUTI89-like IncF plasmids with RST F51:A-:B10. We found this plasmid type was significantly associated with higher ARG carriage. F51:A-:B10 plasmids are known to frequently carry class 1 integrons with *aadA1* [73]. Indeed, the most common class 1 integron structure in this collection was *intI1-aadA1-qacEΔ1-sul1*. This integron is not only found in *E. coli* but also in numerous other bacterial species including *Aeromonas *, *Bacillus *, *Citrobacter *, *Klebsiella *, *Pseudomonas *, *Salmonella* and *Vibrio* [74]. While we found F51:A-B10 to be associated with ARGs, pUTI89-like plasmids (also *senB-cjrABC*+), which were dominated by F29:A-:B10 in our collection, were associated with lower ARG carriage. This is somewhat unsurprising given that this plasmid lineage has been reported to be associated with pan-susceptible ExPEC strains [50].

Despite the positive association of *senB-cjrABC+*/non-pUTI89-like IncF plasmids and ARGs, the overall ARG carriage in this retrospective collection was mostly low, particularly regarding ARGs conferring resistance to antibiotics commonly prescribed for complicated UTIs such as gentamycin, ESBLs and fluoroquinolones. However, ARGs encoding resistance to trimethoprim/sulphonamide were considerable. The majority of ESBL genes were found in ST131, which is unsurprising given that this lineage is thought to have played a central role in the global increase of ESBL-producing *Enterobacteriales* [75]. Though now dominating in Australian ST131 C1 and A clades [56], *bla*_CTX-M-27_ genes were not detected in any of the ST131 isolates from OBH, indicating that this gene may not have yet reached this population. Most of the genotypic fluoroquinolone resistance we observed was attributed to dual *parC* and *gyrA* mutations in ST131 isolates. Interestingly, however, while dual *gyrA*-1AB and *parC*-1aAB were previously thought to be highly specific to ST131 clade C isolates [76], we found these specific mutations in an ST405 isolate. Recently we also reported these mutations in ST131 clade A isolates [56]. Overall, ST131 isolates possessed the most robust MDR profiles, followed by ST10 and ST69 isolates. These STs had the highest prevalence of *intI1*, giving credence to class 1 integrons as reliable markers for MDR [77]. Curiously, while truncated *intI1* genes are common among *E. coli* populations [78] and can be used as epidemiological markers [79], here only 16 % of *intI1* genes had deletions, which may speak to the retrospective nature of this collection, or possibly that truncated *intI1* genes are more prevalent in non-human isolates. For example, in an Australian study of 425 critically drug-resistant *E. coli* from gulls sampled in 2012, 242 (57 %) were determined to carry class 1 integrons. Of these, 64 % showed 3′-truncations in *intI1*, most often associated with IS*26*, with identical truncations found across multiple lineages. Often these truncations are missed by high-throughput gene identification.

Recent studies have shown that rural populations are more likely to receive inappropriate antibiotics for inappropriate durations than urban populations [80, 81]. OBH services regional, rural and remote communities, so one could speculate that the carriage of ARGs and class 1 integrons in UTI isolates would be higher here than in an urban hospital setting. However, the overall *intI1* carriage was lower compared to that reported in a study of UTI isolates from three metropolitan Australian hospitals during the same time period (26.3 % vs 34 %) [82]. One possible explanation for this is that urban surface waters and sediments can have higher ARG loads and plasmid carriage compared to rural samples [83], meaning that the urban environment could be a significant driver for AMR.

UTIs are predominately community-acquired and, beyond the human gastrointestinal tract, the environment, sewage and abattoir waste, retail meats, and animals (wild, food and companion) have all been suggested as ExPEC reservoirs [18, 21, 27, 28, 32, 47, 52, 79]. To explore potential interspecies movement, we performed phylogenetic and SNP analyses on our top four STs (ST73, ST95, ST131, ST127) and compared them to human and non-human-sourced *E. coli* genomes of Australian origin. There is currently no universal SNP threshold to infer *E. coli* transmission, though recent efforts have determined that a 17 SNP cut-off is useful in determining nosocomial outbreaks [84, 85]. Given that we were interested in identifying potential cross-sectoral species transmission, we used a putative SNP threshold of 30. ST73 was previously described as a human-specific ExPEC lineage [86, 87]. However, recent studies have shown that ST73 is prominent in cats [55, 88, 89], dogs [52, 54, 90], killer whales [91] and some avian populations [92]. Consistent with previous studies, our phylogenetic analysis showed ST73 as diverse [53, 93], and that animal-sourced isolates tended to cluster together [54, 55]. Nevertheless, we did identify clusters of very closely related (<30 SNPs) ST73 isolates from human and non-human sources. These low interspecies SNP counts are particularly relevant given the phylogenetic diversity of ST73. We previously showed the potential for interspecies movement in an Australian ST131 population [56], but ST131s have a relatively conserved core genome even amongst the different clades. Conversely, ST73 isolates can differ by thousands of SNPs [93] and indeed we found that some human-sourced ST73 isolates differed from other ST73 human-sourced isolates by up to 5952 SNPs. *E. coli* ST127 has been described as an emerging, highly virulent, human pathogen [67, 94] but has also been isolated from companion animals [32, 52, 54, 55, 88], killer whales [91] and bats [95]. Our investigation did not find any human-sourced ST127 isolates with <30 SNPs from isolates of animal sources. Nevertheless, a recent study of ST127 reported that some isolates from geographically proximal and distal human- vs. companion animal-sources varied by <30 SNPs [32]. *E. coli* ST95 is a prominent cause of both human and avian diseases [96], and the zoonotic potential of this lineage is well documented, particularly in relation to O1:H7 strains [97–99], or those belonging to clade I [18]. The most common serotype in this hospital ST95 population was O1:H7, but these were dispersed predominately among human-dominated clade A, and we found no human-sourced isolate under 30 SNPs from any non-human-sourced isolate.

It is evident that certain STs are more likely to cause UTIs, but the mechanisms behind pathogenesis remain elusive. Congruent with previous studies we found that ST73 was most commonly isolated from patients with cystitis [86], ST95 was associated with sepsis [100] and ST131 was most commonly retrieved from kidney infections [101]. However, we found no significant difference in VAG carriage between isolates from different pathologies. Indeed, we found no significant differences in VAGs, or any other gene, between UTI isolates and non-UTI isolates. Furthermore, we found several examples of isolates with small SNP distances in isolates from patients experiencing different pathologies. Even the molecular definition of UPEC is problematic. By definition, 72 % of our isolates qualified as UPEC but excluded were 75 isolates from patients with UTIs. The majority of ExPEC VAGs encompass siderophore systems, urinary tract-specific adhesins and immune evasion effectors [102], all of which were ubiquitous in our collection. It is important to note that *E. coli* belonging to phylogroup B2, particularly ST73, ST131, ST95 and ST141, are dominant in the gut of healthy humans and their frequency has increased in the commensal faecal population over the past 40 years [103, 104]. These genes prime ExPEC for survival outside of the gastrointestinal tract but also play a role in its persistence in the human gut, while pathogenesis is likely to involve underlying patient factors, such as comorbidities and age [105], or a combination of VAGs. On that note, PAIs can encode several different VAGs and are known to play a major role in the evolution of ExPEC [106]. We observed a correlation between five specific PAIs and STs and found that overall ST127 isolates carried the most PAIs, a feature that was noted in a recent report of ST127 [32].

Our study has some limitations, namely the lack of temporal association between the human clinical and animal isolates, given that the OBH collection is from 2006 and the animal isolates used for comparison were all isolated later (2009–2019). Additionally, AMR phenotypes were not available for the isolates, but ARG presence and resistance phenotypes are typically highly congruent in *E. coli* [107].

## Conclusions

This study describes a phylogenomic analysis of a large cohort of *E. coli* from the urine of patients with uro-centric disease from a major rural hospital in NSW, Australia. While it is unsurprising that the top six STs are the pandemic lineages ST73 > ST95 > ST127 > ST131 > ST12 which accounted for 48 % of all isolates, our study overlays the distribution of F virulence plasmids, PAIs and ARG cargo and provides deeper insights into lineage evolution. The collection is dominated by *E. coli* that carry plasmids with *senB-cjrABC* with those carrying F29:A-:B10 (pUTI89) lacking carriage of antibiotic resistance whereas those carrying *senB-cjrABC* but not closely related to pUTI89 carry a greater antibiotic resistance gene load and were typically *intI1*+. We were able to provide evidence of isolate movements between patients within and across hospital settings, demonstrate the persistence of a clonal lineage from the same patient over short and considerable time periods, and importantly demonstrate evidence of occasional interspecies transmission of ST73, particularly between humans and companion animals. Our study underlines the importance of taking a One Health genomic approach to pathogen surveillance.

## Supplementary Data

Supplementary material 1
